# Cochlear neuropathy and the coding of supra-threshold sound

**DOI:** 10.3389/fnsys.2014.00026

**Published:** 2014-02-21

**Authors:** Hari M. Bharadwaj, Sarah Verhulst, Luke Shaheen, M. Charles Liberman, Barbara G. Shinn-Cunningham

**Affiliations:** ^1^Center for Computational Neuroscience and Neural Technology, Boston UniversityBoston, MA, USA; ^2^Department of Biomedical Engineering, Boston UniversityBoston, MA, USA; ^3^Department of Otology and Laryngology, Harvard Medical SchoolBoston, MA, USA; ^4^Eaton-Peabody Laboratories, Massachusetts Eye and Ear InfirmaryBoston, MA, USA; ^5^Harvard-MIT Division of Health Sciences and Technology, Speech and Hearing Bioscience and Technology ProgramCambridge, MA, USA

**Keywords:** temporary threshold shift, frequency-following response, auditory steady-state response, individual differences, aging, auditory nerve, noise-induced hearing loss, temporal coding

## Abstract

Many listeners with hearing thresholds within the clinically normal range nonetheless complain of difficulty hearing in everyday settings and understanding speech in noise. Converging evidence from human and animal studies points to one potential source of such difficulties: differences in the fidelity with which supra-threshold sound is encoded in the early portions of the auditory pathway. Measures of auditory subcortical steady-state responses (SSSRs) in humans and animals support the idea that the temporal precision of the early auditory representation can be poor even when hearing thresholds are normal. In humans with normal hearing thresholds (NHTs), paradigms that require listeners to make use of the detailed spectro-temporal structure of supra-threshold sound, such as selective attention and discrimination of frequency modulation (FM), reveal individual differences that correlate with subcortical temporal coding precision. Animal studies show that noise exposure and aging can cause a loss of a large percentage of auditory nerve fibers (ANFs) without any significant change in measured audiograms. Here, we argue that cochlear neuropathy may reduce encoding precision of supra-threshold sound, and that this manifests both behaviorally and in SSSRs in humans. Furthermore, recent studies suggest that noise-induced neuropathy may be selective for higher-threshold, lower-spontaneous-rate nerve fibers. Based on our hypothesis, we suggest some approaches that may yield particularly sensitive, objective measures of supra-threshold coding deficits that arise due to neuropathy. Finally, we comment on the potential clinical significance of these ideas and identify areas for future investigation.

## Introduction

A significant number of patients seeking audiological treatment have normal hearing thresholds (NHT), but report perceptual difficulties in some situations, especially when trying to communicate in the presence of noise or other competing sounds (e.g., Hind et al., 2011). Such listeners are typically said to have “central auditory processing disorders”, more recently known simply as “auditory processing disorders” (CAPD/APD; Catts et al., 1996; Chermak and Musiek, 1997), a catchall diagnosis testifying to how little we know about the underlying causes.

In some ways, the fact that having NHTs does not automatically predict good performance in these conditions is not particularly surprising. Audiometric thresholds measure the lowest intensities that a listener can *detect*. In contrast, the ability to *analyze* the content of sound requires a much more precise sensory representation of acoustic features across a large dynamic range of sound intensities. Specifically, current audiometric screenings test the lowest level of sound listeners can hear at various frequencies, but they do not test whether they can make judgments about the spectral or temporal content of the sound, analogous to seeing an eye doctor and being asked whether you can tell that light is present, without worrying about whether or not you can tell anything about the object the light is coming from.

Consistent with the idea that analysis of supra-threshold sound differs amongst NHT listeners, many APD patients seek help precisely because they notice difficulties in situations requiring *selective auditory attention* (Demanez et al., 2003), which places great demands on the auditory system. Moreover, recent laboratory evidence suggests that the prevalence of NHT listeners with APD-like symptoms may be greater than one might predict based on the number of people seeking audiological treatment. Specifically, in the lab, NHT listeners have vastly different abilities on the types of tasks that typically frustrate APD listeners. One recent study shows that when NHT subjects are asked to report spoken digits from one direction amidst otherwise similar speech, performance ranges from chance levels to nearly 90% correct, with the bottom quartile of listeners falling below 60% correct (Ruggles and Shinn-Cunningham, 2011). Crucially, when subjects made errors, they almost always reported a digit coming from a non-target direction rather than an unspoken digit, suggesting that differences were unlikely due to higher-level deficits involving language such as differences in speech intelligibility. Instead, the errors appeared to be due to failing to select the target stream from amidst the maskers. Yet none of the listeners in the study complained of hearing difficulties, even those at the bottom of the distribution; moreover, none had entertained the idea of seeking audiological treatment.

Differences in higher-order processing clearly contribute to individual differences in complex tasks such as the ability to selectively attend, process speech, or perform other high-level tasks (for instance see Surprenant and Watson, 2001). However, in this opinion paper, we focus on how low-level differences in the precision of spectro-temporal coding may contribute to differences in performance. We argue that poor sensory coding of supra-threshold sound is most likely to be revealed in complex tasks like those requiring selective attention, which helps to explain the constellation of symptoms that lead to APD diagnoses. Selective auditory attention hinges on segregating the source of interest from competing sources (object formation; see Bregman, 1990; Darwin and Carlyon, 1995; Alain and Arnott, 2000; Carlyon, 2004), and then focusing on that source based on its perceptual attributes (object selection; see Shinn-Cunningham, 2008; Shinn-Cunningham and Best, 2008). Both object formation and object selection rely on extracting precise spectro-temporal cues present in natural sound sources, which convey pitch, location, timbre, and other source features. Given this, it makes sense that listeners with poor supra-threshold coding fidelity notice problems in crowded social settings, an ability that depends upon robust coding of supra-threshold sound features.

Here, we argue that the fidelity with which the auditory system encodes supra-threshold sound is especially sensitive to the number of intact auditory nerve fibers (ANFs) encoding the input. In contrast, having NHTs likely depends only on having a relatively small but reliable population of ANFs that respond at low intensities. Indeed, one recent study shows that, in animals, audiometric thresholds can be normal even with only 10–20% of the inner hair cells (IHCs) of the cochlea intact (Lobarinas et al., 2013). Our hypothesis is that the convergence of multiple ANFs, while possibly redundant for detecting sound, is critical for analyzing supra-threshold sound.

In this paper, we first consider how supra-threshold sound content is normally encoded, focusing particularly on temporal coding. We then review animal evidence for *cochlear neuropathy*, a reduction in the number of ANFs responding to supra-threshold sound. We argue that this neuropathy can help explain why some listeners have difficulty performing selective attention and other supra-threshold tasks, despite having NHTs. We discuss evidence that lower-spontaneous rate ANFs (lower-SR ANFs; i.e., those with rates below about 18 spikes/s) may be especially vulnerable to damage. We hypothesize that lower-SR ANFs may play a critical role in coding supra-threshold sound features, particularly under challenging conditions. We then discuss the use of the subcortical steady-state response (SSSR) to quantify temporal coding in the early portions of the auditory pathway, including the challenges inherent in interpreting the SSSR and relating it to single-unit neurophysiology. With the help of simple models of brainstem responses, we suggest measures that may emphasize the effect of neuropathy on the SSSR. Using these ideas, we suggest future experiments to (1) test our hypothesis that cochlear neuropathy contributes to the supra-threshold coding deficits seen in some listeners; and (2) develop sensitive, objective correlates of such deficits that may be useful, clinically.

## Coding of supra-threshold sound

### The diversity of auditory nerve fibers

**Figure 1:**
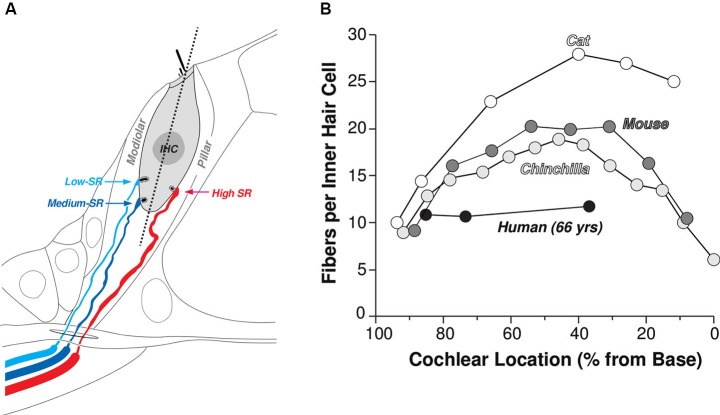
**Innervation of the IHCs by terminals of the cochlear nerve. (A)** Schematic illustrating the spatial separation of the synaptic contacts of high- (SR > about 18 spikes/s) vs. medium- and low-SR fibers on the pillar vs. modiolar sides of the IHCs, respectively. **(B)** Counts of cochlear nerve terminals per IHCs as a function of cochlear location from four mammalian species: cat (Liberman et al., 1990), mouse (Maison et al., 2013), chinchilla (Bohne et al., 1982) and human (Nadol, 1983).

ANFs comprise the sole conduit for information about the acoustic environment, carrying spike trains from the cochlea to the central auditory system. As schematized in Figure 1A, each ANF contacts a single IHC via a single synapse. At each synapse, an electron-dense ribbon sits near the pre-synaptic membrane surrounded by a halo of glutamatergic vesicles. Sound in the ear canal leads to cochlear traveling waves that deflect IHC stereocilia, causing the opening of mechanoelectric transduction channels and a graded change in the IHC membrane potential. At the IHC’s synaptic pole, this sound-driven receptor potential drives an influx of calcium causing an increased probability of fusion of synaptic vesicles with the IHC membrane in the region of the ribbon. Glutamate released into the synaptic cleft binds to the AMPA-type glutamate receptors at the post-synaptic active zone, causing depolarization and action potentials in the ANF.

**Figure 2:**
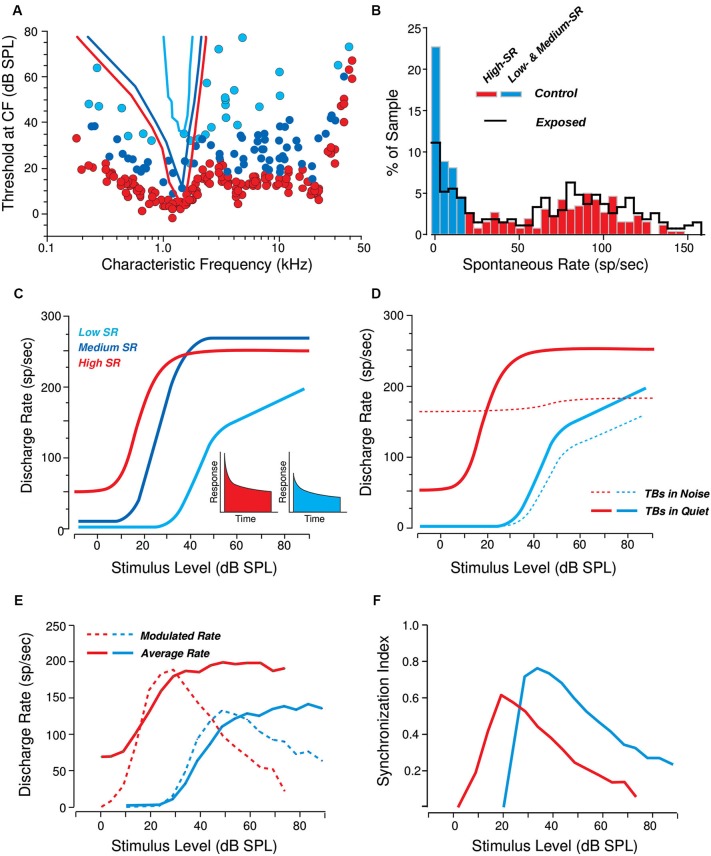
**Response differences among cochlear nerve fibers of the three SR groups. (A)** Threshold tuning curves of example high- medium- and low-SR fibers (see key in **C**) are superimposed on a scatterplot of thresholds at the characteristic frequency (CF) for all the fibers sampled from one animal. Data from cat (Liberman, 1978). **(B)** Distribution of spontaneous rates in large samples of cochlear nerve fibers before (red and blue bars) vs. after (black line) a noise exposure causing a reversible elevation of thresholds. Data from guinea pig (Furman et al., 2013). **(C, D)** Schematic rate-vs-level functions for high-, medium-, and low-SR fibers to tone bursts (TBs) at the CF, in quiet **(C)** and in continuous background noise at a fixed 0 dB spectrum level **(D)**. Data from cat (Liberman, 1978; Costalupes et al., 1984). The insets in panel **C** show schematic peri-stimulus time histograms of the response to a moderate-level tone burst: onset rates are higher in the high-SR fiber than in the low-SR fiber. **(E, F)** Responses to SAM tones in high- vs. low-SR fibers expressed as average rate and modulated rate **(E)** or average synchrony (**F**; see text for definitions). Responses are to carrier tones at the CF, amplitude modulated (AM) at 100 Hz. Data from cat (Joris and Yin, 1992).

Between 10 and 30 ANFs synapse on each IHC, depending on species and cochlear location (Figure 1B), and there are roughly 3500 IHCs along the 35 mm cochlear spiral in humans. Thus, all the information we receive about our acoustic world is carried via the roughly 30,000 ANFs emanating from each cochlea. ANFs in the mammalian inner ear can be subdivided into three functional groups. The classification is based on spontaneous discharge rate (SR; i.e., the spike rate in the absence of sound), because it is easy to quantify, but the key functional differences are in the sensitivity to sound. High-SR fibers have the lowest thresholds, low-SR have the highest thresholds, and medium SR thresholds are intermediate between the two (Figure 2A). The distribution of SRs is fundamentally bimodal (Figure 2B) with roughly 40% in the lower peak (SR < about 18 spikes/second), which includes both low-SR and medium-SR fibers (15% and 25% of all ANFs, respectively) and 60% in the higher peak (Liberman, 1978). In this paper, we shall use the term lower-SR ANFs to refer jointly to the low- and medium-SR groups, which are sometimes distinguished in the literature.

Anatomical studies suggest that all three ANF types can innervate the same IHC, however, lower-SR fibers have thinner axons, fewer mitochondria, and tend to synapse on the modiolar side of the IHC. In contrast, high-SR fibers have thicker axons, more mitochondria, and synapse on the pillar side (Liberman, 1982). There are also systematic differences in the sizes of pre-synaptic ribbons and post-synaptic glutamate-receptor patches (Liberman et al., 2011). All three ANF types send their central axons to the cochlear nucleus (CN), where they branch, sending collaterals to the anteroventral, posteroventral, and dorsal subdivisions. Although branches from all SR types are present in each CN subdivision, low- and medium-SR fibers give rise to more endings than high-SR fibers, especially in the small-cell cap of the anteroventral CN (Ryugo and Rouiller, 1988; Liberman, 1991). Hence, lower-SR fibers may have more downstream influence than suggested by the fact that they make up less than half of the population at the level of the auditory nerve (AN).

The diversity of ANF threshold sensitivity is believed to be important in intensity coding in the auditory system, where level discrimination abilities are near-constant over a range of 100 dB or more (Florentine et al., 1987; Viemeister, 1988). This large dynamic range may be mediated, at least in part, by the differing dynamic ranges of low-, medium-, and high-SR fibers. As represented in Figure 2C, high-SR fibers, whose response thresholds are at or near behavioral detection threshold, likely determine the ability to detect sounds in a quiet environment. However, 20–30 dB above threshold, their discharge rate saturates. By virtue of their higher thresholds and extended dynamic ranges, the lower-SR fibers may be particularly important for extending the dynamic range of hearing. Possibly more important is their contribution to hearing in a noisy environment. Activity of high-SR fibers is relatively easy to mask with continuous noise, as schematized in Figure 2D. Because they are so sensitive to sound, even near-threshold noise increases the background discharge rate of high-SR fibers. This continuous activation causes synaptic fatigue (i.e., vesicle depletion) and thus also decreases their maximum discharge rate to tone bursts or other transient signals that might be present (Costalupes et al., 1984; Costalupes, 1985). By virtue of their higher thresholds, the lower-SR fibers are more resistant to background noise. Thus with increasing levels of continuous broadband masking noise, lower-SR fibers likely become increasingly important to the encoding of acoustic signals, because they will increasingly show the largest changes in average discharge rate in response to transient supra-threshold stimuli (Figure 2D; also see Young and Barta, 1986).

### Temporal coding and its importance for auditory perception

As a result of cochlear filtering, each ANF is driven by a narrow frequency band of sound energy. Thus, the temporal information encoded by the ANFs can be logically separated into two parts; the temporal fine-structure (TFS), corresponding to the timing of the nearly sinusoidal narrowband carrier fluctuations, and the slower temporal envelope of that carrier, whose temporal fluctuations are limited by the bandwidth of the corresponding cochlear filter. For low-frequency cochlear channels, ANFs convey both TFS and envelope information; neural spikes are phase-locked to the carrier and the instantaneous firing rate follows the envelope. At higher frequencies, ANFs do not phase lock to the TFS; however, responses convey temporal information by phase locking to envelope fluctuations.

Although different perceptual attributes of natural sound are encoded by different spectro-temporal cues, many depend on reliable timing information. For instance, the computation of interaural time differences (ITD), important for spatial perception of sound, requires temporal precision on the order of tens of microseconds (Blauert, 1997). While perceptually, TFS information in low-frequencies is the dominant perceptual cue determining perceived location (at least in anechoic conditions; Wightman and Kistler, 1992), for broadband and high-frequency sounds, ITDs can be conveyed by the envelope alone. Moreover, high-frequency envelope ITDs can be perceived nearly as precisely as low-frequency TFS ITDs (Bernstein and Trahiotis, 2002). In addition, envelopes may play a significant role in space perception in everyday settings such as rooms, where reverberant energy distorts TFS cues (Bharadwaj et al., 2013; Dietz et al., 2013). The coherence of the temporal envelope across channels helps to perceptually bind together different acoustic constituents of an “object” in the auditory scene (Elhilali et al., 2009; Shamma et al., 2011). Coding of pitch and speech formants also may rely, at least in part, on both TFS and envelope temporal information, although the precision needed to convey this information is less than that needed to extract ITDs (see Plack et al., 2005 for a review). On an even slower time scale, speech meaning is conveyed by fluctuations in energy through time. Thus, a range of temporal features in both TFS and envelopes are necessary to enable a listener to parse the cacophonous mixture of sounds in which they commonly find themselves, select a sound source of interest, and analyze its meaning. Importantly, almost all of these tasks, when performed in everyday settings, require analysis of temporal information at supra-threshold sound intensities.

To exacerbate matters, everyday settings typically contain competing sound sources and reverberant energy. Both degrade the temporal structure of the sound reaching a listener’s ears, reducing the depth of signal modulations and interfering with the interaural temporal cues in an acoustic signal. If amplitude modulation is weakly coded in a listener with cochlear neuropathy, degradations in the input signal modulations due to competing sound and reverberant energy may render spatial information diffuse and ambiguous, pitch muddy, and speech less intelligible (e.g., see Stellmack et al., 2010; Jørgensen and Dau, 2011). TFS cues convey information important for speech intelligibility in noise (Lorenzi and Moore, 2008). Envelope cues are important for speech-on-speech masking release (Christiansen et al., 2013). Given all of this, a listener with degraded coding of envelope and TFS is most likely to notice perceptual difficulties when trying to understand speech in challenging settings, even if they do not notice any other deficits and have no difficulty in quiet environments. Thus, we hypothesize that differences in the fidelity with which the auditory system encodes supra-threshold TFS and amplitude modulation accounts for some of the inter-subject differences that NHT listeners exhibit in tasks such as understanding speech in noise or directing selective auditory attention (also See Section Human Data Consistent with the Neuropathy Hypothesis). Based on this idea, we argue that a method for measuring supra-threshold temporal coding fidelity may have important clinical applications, enabling quantification of supra-threshold hearing deficits that affect how well listeners operate in everyday environments, but that are not commonly recognized today.

### Consequences of cochlear neuropathy for temporal coding

One consequence of cochlear neuropathy (i.e., a reduction in the number of ANFs conveying sound) will be a reduction in the fidelity of temporal coding of supra-threshold sound. For instance, convergence of multiple, stochastic ANF inputs leads to enhanced temporal precision in the firing pattern of many CN cells (e.g., see Joris et al., 1994; Oertel et al., 2000). Thus, a reduction in the overall number of ANFs will reduce the precision with which both TFS and envelope temporal information are conveyed to higher centers (see also Lopez-Poveda and Barrios, 2013). While the importance of TFS coding for various aspects of sound perception cannot be overstated, we only briefly discuss TFS coding here. We focus primarily on the implications of cochlear neuropathy on the fidelity with which envelope information is conveyed. This focus is motivated particularly by recent data from guinea pigs and mice that suggest that noise-induced neuropathy preferentially damages the higher-threshold, lower-SR cochlear nerve fibers (Furman et al., 2013), rendering envelope coding especially vulnerable, as explained below.

Damage to lower-SR ANFs is likely to be especially detrimental to supra-threshold coding of sound envelopes, as high-SR fibers cannot robustly encode envelope timing cues in sounds at comfortable listening levels. Specifically, the average firing rate of high-SR ANFs (ignoring the temporal pattern of the response) saturates at levels roughly 20–30 dB above threshold, around the sound level of comfortable conversation (see red solid line in Figure 2E). In addition, both measures of phase locking to the envelope (namely the modulated rate, which is the magnitude of the frequency domain representation of the post-stimulus time histogram of the ANF response, evaluated at the fundamental frequency of the input signal; see dashed red line in Figure 2E) and the synchronization index (also known as the vector strength, calculated as the modulated rate normalized by one half of the average rate; see red line in Figure 2F) of high-SR neurons drop off as sound levels approach and exceed comfortable listening levels. This drop off is particularly detrimental for relatively intense sounds with shallow modulation depths, where both the crests and troughs of the envelope of the signal driving the high-SR ANFs fall in the saturation range of intensities, resulting in relatively poor modulation in the temporal response of these fibers (Joris and Yin, 1992). In contrast, lower-SR fibers are more likely to encode these envelope fluctuations because they are likely to be at an operating point where the firing rate (in the steady–state) is still sensitive to fluctuations in the sound level. If noise exposure causes a selective neuropathy that preferentially affects lower-SR fibers, then the ability to analyze envelopes at conversational sound levels is likely to be impaired. Both theoretical simulations and preliminary experimental evidence from envelope following responses (EFRs, described in Section Objective Measures of Subcortical Temporal Coding) recorded in mice and humans are consistent with this reasoning, as discussed in Section Evidence for Cochlear Neuropathy.

### Objective measures of subcortical temporal coding

Many psychophysical studies have been devoted to the development and discussion of behavioral measures to assess temporal coding in both NHT and hearing-impaired listeners (see Moore, 2003; Strelcyk and Dau, 2009). On the other hand, SSSRs provide an objective window into how the subcortical nuclei of the ascending auditory pathway encode temporal information in sound. While behavioral characterizations are important indicators of everyday hearing ability, in order to limit the length and scope of this opinion paper and still provide substantial discussion, here we focus on objective, physiological measures that can quantify the temporal coding precision of supra-threshold sound in the individual listener. Such measures may also be helpful in identifying some of the mechanisms that lead to individual differences in behavioral ability.

SSSRs refer to the scalp-recorded responses originating from subcortical portions of the auditory nervous system. These responses phase lock both to periodicities in the acoustic waveform and to periodicities induced by cochlear processing (Glaser et al., 1976). SSSRs are related to auditory brainstem responses (ABRs; the stereotypical responses to sound onsets and offsets; Jewett et al., 1970); however, whereas ABRs are transient responses to sound onsets and offsets, SSSRs are sustained responses to ongoing sounds that can include responses phase locked to both the fine structure and the cochlear-induced envelopes of broadband sounds. SSSRs have been used extensively in basic neurophysiologic investigation of auditory function and sound encoding (e.g., Kuwada et al., 1986; Aiken and Picton, 2008; Gockel et al., 2011; also see Krishnan, 2006; Chandrasekaran and Kraus, 2010, for reviews). Given the frequency specificity possible with SSSRs, they have also been proposed as a potential tool for objective clinical audiometry (Lins et al., 1996). In addition, SSSRs have been shown to be sensitive to deafferentation in that IHC loss leads to degraded SSSRs, especially at moderate sound levels (Arnold and Burkard, 2002).

While there are many studies of SSSRs, confusingly, different branches of the scientific literature use different names to refer to the same kinds of measurements. Periodic responses to amplitude-modulated sounds originating from both the sub-cortical and cortical portions of the auditory pathway are often collectively referred to as auditory steady-state responses (ASSRs) (Galambos et al., 1981; Stapells et al., 1984; Rees et al., 1986). However, brainstem SSSRs can be distinguished from responses generated at the cortical level by virtue of their relatively high frequency content; practically speaking, cortical and SSSR responses can be extracted from the same raw scalp recordings by appropriate filtering (e.g., see Krishnan et al., 2012; Bharadwaj and Shinn-Cunningham, 2014). The responses that specifically phase lock to the envelope of amplitude modulated (AM) sounds have been referred to as EFRs or amplitude modulation following responses (AMFRs; Dolphin and Mountain, 1992; Kuwada et al., 2002). In the recent literature, SSSRs are most commonly referred to as frequency following responses (FFRs), a term originally used to denote responses phase locked to pure tones (Marsh et al., 1975). Since the term FFR hints that responses are phase locked to the acoustic frequency content of input sound (i.e., the fine-structure of narrowband or locally narrowband sounds), here we will use the term “SSSR” to describe the sustained responses originating from subcortical portions (at frequencies >80 Hz or so in humans) of the auditory pathway. More specifically, we will focus on EFRs: SSSRs that are locked to the envelope.

While EFRs provide a convenient non-invasive measure of subcortical envelope coding, there are several difficulties in interpreting them. First, they represent neural activity that is the sum of a large population of neurons, filtered by layers of brain tissue, skull, and scalp. Depending on the stimulus parameters, thousands of neurons in each of multiple subcortical nuclei may contribute to the EFR (Kuwada et al., 2002). Neurons from several regions along the tonotopic axis could contribute to the EFR for high-level sounds due to spread of excitation, even for narrow-band sounds. Thus, relating EFR results to physiological responses of single neurons is not straightforward. ANF modulation frequency responses are uniformly low pass; high characteristic frequencies (CFs) fibers (>10 kHz) have cutoff frequencies around 1 kHz in cat (Joris and Yin, 1992). Below 10 kHz, cutoff frequency is dependent on CF, suggesting a limit imposed by an interaction between the content of the input signal and the bandwidths of cochlear filters (Joris and Yin, 1992). As signals ascend the auditory pathway, they are transformed from a temporal to a rate code, with the upper limit of phase locking progressively shifting to lower modulation frequencies (summarized in Figure 9 of Joris et al., 2004; see also Frisina et al., 1990; Joris and Yin, 1992; Krishna and Semple, 2000; Nelson and Carney, 2004). Modulation frequencies in the 70 to 200 Hz range elicit phase-locked responses in a cascade of subcortical auditory structures, from cochlear hair cells to inferior colliculus (IC) neurons, suggesting that many sources can contribute to the EFRs in this frequency range. Luckily, compared to the IC, the more peripheral EFR generators generate relatively weak responses, both because they drive smaller synchronous neural populations and because they are more distant from the measurement site.

Based on single-unit data, reversible inactivation studies, irreversible lesion studies, and studies analyzing EFR group delay, it has been argued that the dominant generators of the EFR move from caudal (AN and CN) to rostral (inferior colliculus or IC) as modulation frequency decreases (Sohmer et al., 1977; Dolphin and Mountain, 1992; Kiren et al., 1994; Herdman et al., 2002; Kuwada et al., 2002). These studies provide evidence that the IC dominates EFRs at modulation frequencies between about 70 and 200 Hz, in all species tested. Changes in the slope of the response phase vs. input modulation frequency can be used to calculate apparent latency of the sources and thereby infer changes in the relative strengths of different neural generators in the mixture (Kuwada et al., 2002); regions where the slope is constant indicate regions where the mixture of generators is constant. Above 200 Hz, the pattern of these changes varies across species, probably due to differing head sizes and shapes. Humans, rabbits, and mice exhibit regions of constant phase slopes out to 500, 700, and 1000 Hz, respectively (Kuwada et al., 2002; Purcell et al., 2004; Pauli-Magnus et al., 2007); in contrast, in gerbils, the phase slopes above 200 Hz are not constant (Dolphin and Mountain, 1992). These differences in phase slopes indicate that the specificity of EFRs is species-dependent. However, in all species it is clear that manipulation of modulation frequency can be used to bias responses towards more rostral or more caudal sources.

Despite these complications, all acoustic information is conveyed to the brain through the ANFs; moreover, deficiencies at the level of the ANF can be expected to have an effect downstream, in higher-order processing centers. Therefore, EFRs originating in the brainstem/mid-brain are likely to reflect the consequences of ANF neuropathy. Indeed, by using different stimuli, it may be possible to emphasize the contribution of different subcortical sources (by changing the modulation frequency of the input) or different portions of the cochlear partition (by changing the acoustic carrier of the signal). In particular, metrics such as the phase-locking value (PLV) can be calculated to quantify the robustness of temporal coding in the EFR, akin to using the vector-strength to assess temporal coding in single-unit physiology studies (Joris et al., 2004).

**Figure 3:**
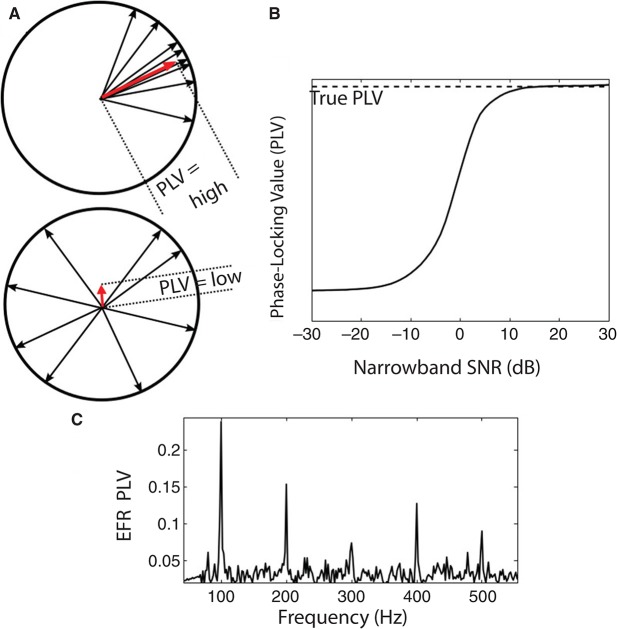
**(A)** An illustration of the PLV metric computation. The SSSR from each trial is represented by a vector (phasor, shown as a black arrow) with unit magnitude and with phase equal to the EFR phase at the frequency bin of analysis. The vector average of these phasors is computed; the magnitude of the resultant vector (shown as red arrow) yields the PLV. The top panel is an example with high PLV: the phase of the responses varies over a narrow range across trials. The bottom panel is an example with low PLV: response phase relative to stimulus onset is essentially random over the unit circle. **(B)** Relationship between the single-trial SNR of the measurement in the frequency bin of interest and the estimated PLV for a simulated signal in additive noise. At sufficiently high SNR values, the estimated PLV converges to the true PLV (aside from a small sample bias that depends on the number of trials). At lower SNRs, the estimate is biased to be lower than the true value. This is an important consideration when comparing PLVs across sound levels or individuals, since the SNR depends on the magnitude of the true underlying response, the geometry of the generators, and the volume conductor in between. **(C)** Sample PLV spectrum obtained in response to a 100 Hz transposed tone at a carrier frequency of 4 kHz at 65 dB SPL (RMS). Strong peaks are evident in the PLV at multiples of the envelope frequency.

When analyzing the temporal precision of signals, the PLV has a straightforward interpretation. The details of the PLV computation and its statistical properties are described in a number of previous studies (e.g., see Lachaux et al., 1999; Bokil et al., 2007; Ruggles et al., 2011; Zhu et al., 2013). Briefly, the PLV quantifies the consistency of the response phase across repetitions of the stimulus presentation (“trials”). For a given frequency bin, the response to each trial can be represented as a unit vector (phasor) in the complex plane whose phase equals the response phase. The PLV then equals the magnitude (length) of the vector average of the phasors, averaged across trials (Figure 3A). If the response is consistently at or near a fixed phase, then the resulting average has a magnitude near one and the PLV is high (top panel, Figure 3A). On the other hand, if the response phase relative to the stimulus is random over the unit circle, the phasors cancel, the resultant vector has a small magnitude, and the PLV is near zero (bottom panel of Figure 3A). An example of the PLV spectrum (computed for EFRs from 400 repetitions of a 100 Hz transposed tone at a carrier frequency of 4 kHz and 65 dB SPL) is shown in Figure 3C. Strong peaks are evident at the fundamental and harmonic frequencies of the envelope. The PLV thus is one way of assessing the temporal coding fidelity of the EFR, and of subcortical encoding of supra-threshold sound.

## Evidence for cochlear neuropathy

### Neuropathy and selective loss of lower-spontaneous discharge rate fibers in animals

Recent studies in both mice and guinea pigs show that noise exposure that causes a *temporary* increase in threshold sensitivity (e.g., initial threshold elevations of as much as 40 dB that completely recover over 3–5 days) nevertheless can cause a rapid loss of 40–50% of the ANF synapses on IHCs as well as a slow death of the ANF cell bodies (spiral ganglion cells) and central axons (Kujawa and Liberman, 2009; Maison et al., 2013). Despite the extent of effects of such exposure on synapses and ganglion cells, it does not typically cause any loss of hair cells. Single-unit recordings in the guinea pig indicate that this noise-induced loss is selective for lower-SR fibers (Furman et al., 2013). Pharmacological studies suggest that this neuropathy is the result of a type of glutamate excitotoxicity, brought on by glutamate overload at particularly active synapses (Pujol et al., 1993). In the central nervous system, glutamate excitotoxicity is mediated by an increase in intracellular calcium concentration (Szydlowska and Tymianski, 2010). Since mitochondria comprise an important intracellular calcium buffering system, the relative paucity of mitochondria in the lower-SR fibers (Liberman, 1980) may contribute to their special vulnerability to glutamate excitotoxicity caused by noise exposure.

In aging mice, there is a steady degeneration of ANFs. Indeed, 30–40% of IHC synapses are lost by roughly 3/4 of the lifespan, an age at which threshold elevation is modest (typically less than 10 dB), but there is no significant loss of hair cells (Sergeyenko et al., 2013). Previous neurophysiological studies of age-related hearing loss in the gerbil suggest that this neurodegeneration is also selective for lower-SR fibers (Schmiedt et al., 1996). Unfortunately, relatively little is known about how aging impacts ANF synapses in humans. The only study that counted IHC synapses in the human inner ear (Figure 1B) found relatively low numbers of IHC synapses; however, this low count may reflect a significant degree of age-related neuropathy rather than a species difference, given that the tissue was obtained from a relatively old individual (63 years of age). Indeed, counts of spiral ganglion cells in an age-graded series of human temporal bones show degeneration of 30%, on average, from birth to death, even in cases with no hair cell loss (Makary et al., 2011). The marked delay between synaptic death and spiral ganglion cell death (1–2 years in mouse, and possibly much longer in humans) suggests that the loss of cochlear nerve synapses on IHCs is almost certainly significantly greater than 30%, on average, in the aged human ear.

Considering that only a small number of sensitive, intact ANFs may be needed for detection in quiet (Lobarinas et al., 2013), it seems likely that even considerable neuropathy would not change thresholds for tones in quiet, and thus would not be detected by standard threshold audiometry. This is even more likely the case if the neuropathy is selective for ANFs with higher thresholds, which are not active near perceptual thresholds. It also seems likely that a loss of a large population of high-threshold ANFs could dramatically affect auditory performance on complex tasks that require analysis of supra-threshold sound content, such as those requiring the extraction of precise timing cues or extracting a signal in a noisy environment, as discussed above. Thus, we hypothesize that cochlear neuropathy in general—and possibly selective neuropathy of high threshold fibers in particular—is one of the reasons that aging often is found to degrade human performance on tasks requiring analysis of the content of supra-threshold sound.

### Human data consistent with the neuropathy hypothesis

**Figure 4:**
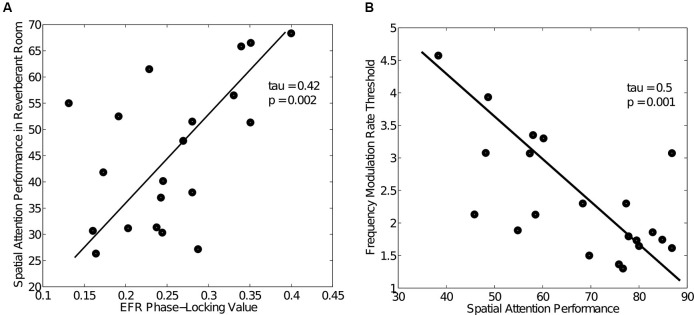
**Human behavioral and EFR data (data from Ruggles et al., 2011, 2012) showing large variability in both performance and temporal coding fidelity among NHT participants. (A)** Relationship between spatial attention task performance in reverberation and EFR PLV across NHT listeners. Task performance varied from chance levels (30%) to about 70% with a concomitant variation in EFR phase locking. Listeners with good temporal coding of envelopes as measured by the EFR PLV were able to spatially segregate the competing speech streams and performed well. **(B)** Relationship between spatial attention task performance and frequency modulation (FM) detection thresholds (data from Ruggles et al., 2011), a task known to rely on robust encoding of TFS.

While there is no human data yet to directly support the neuropathy hypothesis, a series of studies from our lab are consistent with the hypothesis that cochlear neuropathy causes difficulties with coding of supra-threshold sound for humans and accounts for some of the individual variability seen in listeners with normal audiometric thresholds. NHT listeners exhibit marked differences in how well they can utilize precise temporal information to direct selective attention, from near-chance levels to almost perfect performance (Ruggles and Shinn-Cunningham, 2011). As discussed in Section Consequences of Cochlear Neuropathy for Temporal Coding, cochlear neuropathy could result in degraded coding of both TFS and envelope information. In line with this hypothesis, differences in EFR phase locking accounts for some of this inter-subject variability in performance. Figure 4A shows the relationship between performance in a spatial attention task in reverberation and the PLV calculated from EFRs obtained separately (data from Ruggles et al., 2011, 2012). Pooled over age groups, listeners with higher EFR phase locking performed better in the selective attention task (Kendall *tau* = 0.42, *p* < 0.002). Though age by itself did not correlate with performance in anechoic conditions, when temporal cues in the acoustic mixture were degraded by adding reverberation, middle-aged listeners showed a bigger drop in performance than younger listeners (Ruggles et al., 2012), as if timing cues are encoded less robustly in middle-aged listeners than in young adults. In addition, as shown in Figure 4B, performance also correlated with thresholds for low-rate frequency modulation (FM) detection, a task known to rely on robust temporal coding of TFS (Kendall *tau* = 0.5, *p* = 0.001, data from Ruggles et al., 2011, 2012). Crucially, all listeners in these studies had pure-tone audiometric thresholds of 15 dB HL or better at octave frequencies between 250 Hz and 8 kHz. The small differences in hearing threshold (within the NHT range) that did exist were not correlated with selective attention performance; similarly, reading span test scores (a measure of cognitive ability) were unrelated to performance. These results suggest that both TFS and envelope cues are important in everyday listening under challenging conditions, since individuals with poor TFS and envelope coding (as measured by FM detection thresholds and EFR phase locking respectively) perform poorly in a spatial attention task. (For a complete description of the spatial attention task, the FM detection task and the EFR measures, see Ruggles et al., 2011, 2012).

Several other studies have reported that some listeners with normal thresholds (particularly older participants) perform poorly on certain behavioral tasks, sometimes even on par with hearing-impaired subjects. Yet other studies show that temporal processing of both TFS and envelope degrades with aging and manifests independently of hearing loss (see Fitzgibbons and Gordon-Salant, 2010 for a review). In NHT listeners, sensitivity to ITD varies greatly across the population, with some listeners performing as poorly as older hearing-impaired subjects (see Grose and Mamo, 2010; Strelcyk and Dau, 2009). Recent studies have also demonstrated abnormal speech processing among hearing-impaired listeners even when the frequency content of the speech was limited to regions where thresholds are normal, pointing towards supra-threshold coding deficits (Horwitz et al., 2002; Lorenzi et al., 2009; Léger et al., 2012).

Older listeners also have been shown to exhibit deficits specific to envelope processing across a range of tasks, including speech recognition in the presence of modulated noise maskers (Dubno et al., 2003; Gifford et al., 2007) and temporal modulation sensitivity (Purcell et al., 2004; He et al., 2008). Consistent with this, the highest modulation frequency to which EFRs exhibit phase locking decreases with age (Purcell et al., 2004; Leigh-Paffenroth and Fowler, 2006; Grose et al., 2009), supporting the hypothesis that the robustness of supra-threshold modulation coding is reduced with aging. Using measures of both gap detection and word recognition on a sizeable cohort of young and old listeners, Snell and Frisina (2000) concluded that age-related changes in auditory processing occur throughout adulthood. Specifically, they concluded that deficits in temporal acuity may begin decades earlier than age-related changes in word recognition. Though not direct evidence that neuropathy causes these perceptual difficulties, these results are consistent with our hypothesis, especially given animal data suggesting that both aging and noise-exposure degrade ANF responses (especialy lower-SR fibers) and degrade supra-threshold temporal coding without affecting thresholds (Schmiedt et al., 1996; Kujawa and Liberman, 2009; Lin et al., 2011; Furman et al., 2013). If neuropathy underlies deficits in temporal encoding that predict behavioral differences, it may be possible to develop even more sensitive physiological metrics to capture an individual listener’s supra-threshold coding fidelity. Section Diagnosing Cochlear Neuropathy is devoted to the discussion of this idea.

## Diagnosing cochlear neuropathy

The degree of deafferentation in cochlear neuropathy can be studied directly in animals using invasive methods in combination with histological evaluation, or in humans using post-mortem studies (e.g., Halpin and Rauch, 2009; Makary et al., 2011). However, assessment in behaving humans must be non-invasive, and therefore must employ indirect methods. Given that neuropathy should impact supra-threshold temporal coding, individual behavioral assessment of envelope and TFS coding of sound at comfortable listening levels may prove useful in assessing neuropathy. In order to expose supra-threshold deficits and individual differences, selective attention tasks in adverse conditions (e.g., in a noise background or in a complex, crowded scene) may be most effective. However, given that aging and noise exposure cause outer hair cell loss, elevated thresholds, and other (much-studied) effects, assessment of cochlear function is necessary to ensure that supra-threshold deficits are attributable to neuropathy. Measures of brainstem temporal coding, like the ABR and SSSR, may be helpful in assessing neuropathy objectively and passively; exploring these metrics at high sound levels and low modulation depths (which stresses coding of modulations akin to those important when listening in a crowded scene) may be particularly useful (see Section Emphasizing the Contribution of Lower-Spontaneous Discharge Rate Auditory Nerve Fibers to the Envelope Following Responses). In order to develop and interpret effective, sensitive tests using these types of non-invasive physiological measures, quantitative models that provide testable predictions will be vital. In this section, we consider some of these points, with a focus on objective measures.

### Measuring brainstem coding: auditory brainstem responses vs. subcortical steady-state responses

In animal work, the preferential loss of higher-threshold (lower-SR fibers) leads to a decrease in the supra-threshold growth of the amplitude of wave I of the ABR, without a change in ABR threshold (Kujawa and Liberman, 2009; Furman et al., 2013). In both noise-exposed mice and noise-exposed guinea pigs, the proportional decrement in the magnitude of wave I at high levels (i.e., 80 dB SPL) closely corresponds to the percentage of loss of auditory-nerve synapses. However, by limiting the analysis to animals without permanent threshold shifts in the noise-exposed ear, these experiments remove the confound that changes in hearing threshold are likely to affect wave I amplitude; by design, the supra-threshold changes in ABR amplitude found in these experiments cannot be due to differences in threshold sensitivity, but instead reflect differences in the number of fibers responding to supra-threshold sound. Even in populations with normal thresholds, inter-subject variability in ABR amplitudes complicates analysis. One past study showed that in age- and gender-matched mice, the variance in normal ABR amplitude measures is relatively low (Kujawa and Liberman, 2009); however, the mice in this study were genetically identical. In age- and gender-matched guinea pigs, the variance in ABR amplitude is significantly higher. In the genetically heterogeneous guinea pigs, neuropathy-related changes in ABR amplitude are revealed clearly only when data are analyzed within subject, measuring the effects of noise exposure by normalizing the post-trauma amplitude responses by the responses from the same ear before exposure (Furman et al., 2013). Of course, such a before-and-after approach is unlikely to prove useful for human clinical testing, except in extraordinarily rare circumstances.

The above studies suggest that the ABR may be useful for assessing neuropathy. However, there are a number of reasons why the electrophysiological responses to an AM carrier tone, i.e., the EFR, might be better suited to the assessment of lower-SR neuropathy than the ABR. For one thing, ABR wave I, generated by tone pips, is proportional to the size of the onset responses in the AN. Since, as schematized in Figure 2C, the onset responses of lower-SR fibers are small compared to high-SR fiber onset responses (Taberner and Liberman, 2005; Buran et al., 2010), they make a relatively small contribution to the total onset response, rendering the metric fairly insensitive to the integrity of the lower-SR population. In contrast, the steady-state rates of the three SR groups are of more similar magnitude; a loss of lower-SR fibers should thus cause a greater change in steady-state measures like the SSSR or EFR than transient responses like the ABR. Furthermore, as noted above (see Figure 2F), lower-SR ANFs synchronize more tightly to the envelope of an AM tone than their high-SR counterparts, especially at moderate and high sound intensities (Johnson, 1980; Joris and Yin, 1992). Synchronization in response to AM-tones can be assessed both by the modulated rate (the amplitude of the peri-stimulus time histogram at the stimulus modulation frequency) and synchronization index (or vector strength; see Joris et al., 2004 for a discussion about different measures of envelope coding). The synchronization index of lower-SR fibers can be larger than that of high-SR fibers of similar best frequency. Indeed, preliminary results suggest that in noise-exposed mice, amplitude decrements in EFR responses to an amplitude-modulated carrier tone presented at the frequency region of maximum cochlear neuropathy are a more sensitive measure of deficit than decrements in ABR wave I amplitude (Shaheen et al., 2013). Perhaps more importantly, a phase-based analysis like the PLV can be used to analyze EFR strength, which can be a more robust and more easily interpreted metric than amplitude measures of these far-field potentials, which have a weak signal-to-noise ratio (SNR) and depend on factors such as tissue and head geometry.

### Emphasizing the contribution of lower- spontaneous discharge rate auditory nerve fibers to the envelope following response

As previously discussed (Section Temporal Coding and Its Importance for Auditory Perception), one likely consequence of cochlear neuropathy is a reduction in the fidelity of temporal coding in the brainstem. The idea that cochlear neuropathy may preferentially target lower-SR fibers (Schmiedt et al., 1996; Furman et al., 2013) may be exploited to devise EFR measures that are more likely to capture the effects of neuropathy. Focusing on responses to high-frequency envelopes could prove to be an effective way to assess neuropathy, because envelope fluctuations cannot drive saturated high-SR fibers effectively. Even for “transposed tones” (a modulated high-frequency signal whose envelope mimics the rectified sinusoidal drive of a low-frequency tone operating at low-frequency portions of the cochlea; see van de Par and Kohlrausch, 1997), phase locking of high-SR fibers is reduced at mid to high sound levels (Dreyer and Delgutte, 2006). This effect is likely to be particularly strong for a relatively high-intensity modulated signal with a shallow modulation depth. For such signals, the input intensity of the driving signal will fall within the saturation range of high-SR fibers at all moments; the only fibers that could encode the shallow modulations are the lower-SR fibers. Thus, measures of EFR phase locking to high-frequency, high-intensity, amplitude-modulated signals with shallow modulation may be especially sensitive when assessing lower-SR-fiber status.

**Figure 5:**
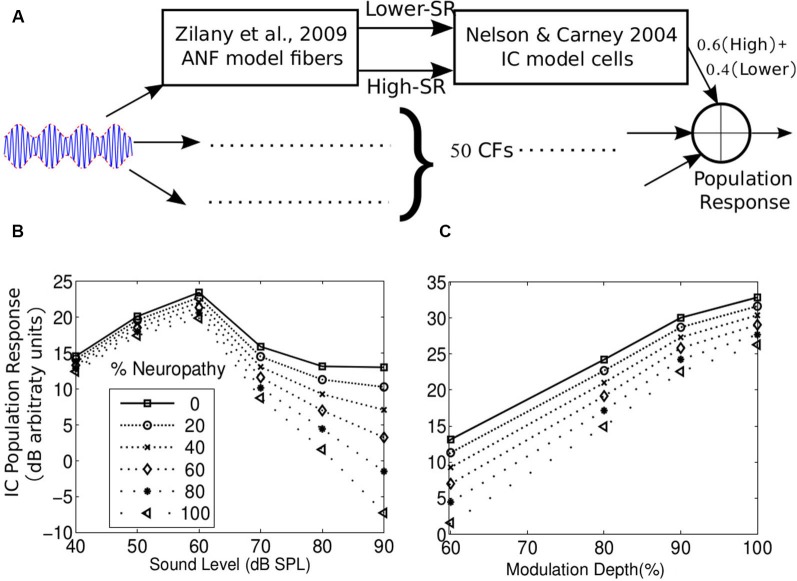
**(A)** A parsimonious model of the population response of IC cells to envelope fluctuations. The model comprised of ANFs (simulated using the Zilany et al., 2009 model) driving the cochlear nucleus (CN), which in turn drives the IC. CN and IC processing of envelope were simulated using the Nelson and Carney (2004) model. A tonotopic array of 50 CFs was used. High- and lower-SR ANFs were simulated at each CF and the corresponding IC responses were combined with weigths equal to the proportion of each group in the population (60% High- and 40% Lower-SR, Liberman, 1978). Neuropathy was simulated by reducing the weight given to the lower-SR driven response. **(B)** Level curves for the population response with different levels of neuropathy for a 100 Hz SAM tone at 4 kHz, with a 60% modulation depth and added broadband noise with a notch centered around 4 kHz and 800 Hz wide on each side. The SNR was fixed at 20 dB (broadband RMS) at all levels. The differences between the levels of neuropathy are most accentuated in the population response at higher stimulus levels. This also suggests that slopes of the level curve at high levels may reflect the level of neuropathy. **(C)** Population response as a function of modulation depth for different levels of neuropathy for an 80 dB SPL SAM tone in notched noise (SNR = 20 dB broadband RMS). The differences between the levels of neuropathy are more evident for smaller modulation depths. In addition, this suggests that the slope of the population response strength as a function of modulation depth may be sensitive to the level of neuropathy.

Here, we use a simple model of brainstem responses to illustrate why EFRs to shallow amplitude modulations and high sound levels are likely to emphasize the contribution of lower-SR fiber responses to the measurements. Given that EFR responses reflect responses at the level of the brainstem/midbrain, likely the IC, we built a model of IC responses (Figure 5A) by combining an established model of the ANF responses (Zilany and Bruce, 2006; Zilany et al., 2009) with previous phenomenological models of amplitude-modulation processing in the IC (Nelson and Carney, 2004). Updated, humanized, ANF model parameters were used for the simulation (Zilany et al., 2014). This model has been shown to predict ANF single-unit envelope response data quite well (Joris and Yin, 1992). Considering that the simulations included stimuli with high sound levels (as in Dau, 2003; Rønne et al., 2012), a tonotopic array of ANFs (and corresponding IC cells) were included to allow for off-frequency contributions. ANFs with 50 CFs uniformly spaced along the basilar membrane according to a place-frequency map were simulated. For each CF, lower- and high-SR fibers were simulated. In order to obtain a population response at the level of the IC, responses to IC cells driven by lower- and high-SR ANFs were averaged with weights proportional to known population ratios (40% Lower-SR fibers and 60% high-SR fibers, see Liberman, 1978). At the level of the IC, the resulting population response is treated as a proxy for the signal driving the EFR. Responses were simulated for a sinusoidally amplitude modulated (SAM) tone with a carrier frequency of 4 kHz and a modulation frequency of 100 Hz. In order to attenuate the contribution of off-frequency neurons to the population response, a broadband noise masker with a notch centered at 4 kHz and extending 800 Hz on either side was added to the SAM tone, as can be done with real EFR measurements in the laboratory. The SNR for the simulations was fixed at 20 dB (broadband root mean square (RMS)). The IC model parameters were set to the values used in Nelson and Carney (2004), which ensured that the 100 Hz modulation frequency was within the band-pass range of the IC cells. Neuropathy was simulated by progressively attenuating the weights given to the IC population driven by lower-SR ANFs, leaving the high-SR population unchanged.

Figure 5 shows the absolute population response magnitude following the 100 Hz modulation in logarithmic units. Results are shown for different amounts of neuropathy, both for different stimulus levels (Figure 5B) and for different modulation depths (Figure 5C). As seen from the figures, neuropathy has the greatest effect on the population response for stimuli at mid to high sound levels and relatively low modulation depths. This is consistent with the idea that the modulated firing rate of high-SR ANFs is drastically attenuated at moderate to high sound levels and low-modulation depths (Joris and Yin, 1992; Dreyer and Delgutte, 2006). Similar results were obtained (not shown) presenting “transposed” tones to this model as well as when using the Rønne et al. (2012) model, where the EFR is obtained by convolving the ANF population response with a “unitary-response” that is designed to aggregate and approximate all transformations of the ANF population response before being recorded in the EFR. In both model approaches, lower- and high-SR ANF driven IC responses were summed linearly to generate the population response. When the lower- and high-SR ANF responses were mixed non-linearly using a coincidence detection process (i.e., a geometric average instead of an arithmetic average) before being delivered to the IC model, the effects of the lower-SR fiber neuropathy were even larger (not shown).

This analysis supports the idea that EFR responses to shallow amplitude modulation at high levels may provide a sensitive, objective correlate of neuropathy. Apart from emphasizing the contribution of lower-SR ANFs, high sound levels are more likely to reveal differences in the number of intact ANFs even if neuropathy is not specific to lower-SR fibers because larger populations of ANFs are recruited overall. These results are also consistent with the report that the ABR wave I amplitude in noise-exposed mice closely corresponds to the amount of neuropathy when the sound level is high (80 dB, Furman et al., 2013) as well as preliminary data from our lab that suggest that individual differences in the EFR are largest at high stimulus levels (Bharadwaj et al., 2013). In addition, inspection of Figure 5B, C suggests that the sizes of the change (i.e., slopes) in the population response with level and with modulation depth both reflect the level of neuropathy. Thus, either of these changes, along with behavioral measures, could be used to assess the ability of the listener to process supra-threshold sound. However, in practice, manipulating modulation depth with the level fixed at a high value may lead to more easily interpreted results than measuring how the EFR changes with overall level (see Section Using Envelope Following Responses to Assess Supra-threshold Coding Fidelity). As explained above, we suggest that individual listeners with normal audiometric thresholds could differ in the number of intact ANFs due to differences in noise exposure, genetic predisposition to hearing damage, and other factors. Given the already-discussed importance of supra-threshold temporal coding for operating in everyday social settings (understanding speech in noise, directing selective auditory attention, etc.), assessment of neuropathy by measurement of EFRs may have a place in audiological practice, especially because such measures are objective and can be recorded passively (making them suitable for use with special populations in which behavioral assessment is not easy).

### Isolating cochlear neuropathy

As noted above, in order to assess neuropathy, it is critical to rule out or otherwise account for cochlear dysfunction. One of the most basic characteristics of cochlear function is the frequency selectivity of the basilar membrane (BM). BM frequency selectivity is correlated with cochlear gain at low sound levels (Shera et al., 2002, 2010) and typically decreases with hearing impairment. BM frequency selectivity can be estimated psychophysically (Patterson, 1976; Glasberg and Moore, 1990; Oxenham and Shera, 2003); however, it is possible that such measures may include small contributions from extra-cochlear factors (such as neuropathy). Alternatively, distortion product otoacoustic emissions (DPOAEs) in response to fixed-level primaries (DPgrams; e.g., see Lonsbury-Martin and Martin, 2007) can be used to assess cochlear function. Because OAEs are generated within the cochlea as a consequence of outer-hair-cell activity and do not depend on afferent processing, measuring them may be preferable to measuring psychophysical tuning curve measures. Specifically, normal DPgrams can be used to establish that poor supra-threshold coding arises post transduction (e.g., via cochlear neuropathy) rather than from outer-hair-cell loss or other problems with cochlear amplification (an approach taken in the animal studies of Kujawa and Liberman, 2009; Furman et al., 2013). To test that cochlear compression is intact at the frequencies tested, either stimulus-frequency OAEs (SFOAEs; Schairer et al., 2006) or DPOAE growth functions can be used (Kummer et al., 1998; Neely et al., 2003). DPOAE suppression tuning curves (Gorga et al., 2011; Gruhlke et al., 2012) or SFOAE phase gradients at low stimulus levels (Shera et al., 2002) can provide estimates of cochlear filter tuning. Henry and Heinz (2012) recently demonstrated the importance of considering differences in cochlear function in order to interpret differences in measures of temporal coding fidelity properly. As this work shows, establishing that participants have normal cochlear sensitivity by measuring both OAEs and audiometric thresholds is crucial when trying to attribute individual differences in SSSRs and psychoacoustic measures to deficits in supra-threshold coding of sound due to neuropathy.

## Future experiments

A growing body of evidence suggests that (1) NHT listeners vary significantly in how well their auditory systems encode supra-threshold sound; and (2) Noise exposure and aging can lead to considerable amounts of neuropathy without affecting audiometric thresholds. We have argued that cochlear neuropathy in general, and selective neuropathy of lower-SR ANFs in particular, may help explain some of the supra-threshold differences in NHT listeners. Although we believe that the diversity of evidence consistent with this hypothesis is compelling, further experiments are necessary to truly establish these ideas and to understand potential implications for audiological practice. Here, we propose a few key areas that we believe merit future investigation.

### Accounting for individual differences in cochlear function

As discussed in Section Isolating Cochlear Neuropathy, experiments seeking to implicate cochlear neuropathy in human perception must account for individual differences in cochlear processing. There are a number of objective metrics of cochlear health including DPOAE and SFOAE growth functions (Kummer et al., 1998; Schairer et al., 2006), DPOAE suppression tuning curves (Gorga et al., 2011; Gruhlke et al., 2012), and SFOAE group delay measurements (Shera et al., 2002; Shera and Bergevin, 2012). However, there are practical concerns that may limit the utility of many of these methods. For instance, using OAE methods to study neuropathy in patients with elevated hearing thresholds may be difficult, as SFOAE amplitudes critically depend on cochlear gain (Shera and Guinan, 1999). DPOAE methods depend more on cochlear compression, rather than cochlear gain (Shera and Guinan, 1999), and thus may prove to be a more robust method for assessing contributions of cochlear function to perception in heterogeneous subject populations (Gruhlke et al., 2012). Experiments are needed to determine what tests best quantify cochlear function, enabling such factors to be teased out when appraising cochlear neuropathy, and developing such tests into clinically useful tools.

### Developing quantitative models of envelope following response generators

Because any human measurements of EFRs only indirectly reflect the responses of ANFs, quantitative models of the subcortical generators of the measured response are critical for understanding results and using them to quantify supra-threshold envelope coding. Data suggest that EFRs primarily reflect responses from the mid-brain, and are dominated by responses in the IC (Smith et al., 1975; Sohmer et al., 1977; Dolphin and Mountain, 1992; Kiren et al., 1994; Herdman et al., 2002). However, further experiments are needed to assess if current physiological models capture the behavior of real EFRs. When applied to modulated high-frequency sounds, simple models of IC responses predict a graded loss in the population response with cochlear neuropathy (see Figure 5), consistent with the idea that the observed heterogeneity of EFR responses in NHT subjects reflects, in part, differences in ANF survival. Instead of modeling individual neurons, others have modeled brainstem responses (ABRs and FFRs) directly using a kernel method (e.g., Dau, 2003; Rønne et al., 2012). In this approach, all subsequent transformations of the AN responses are modeled by a linear system approximation; model AN responses are used to deconvolve click-ABRs to obtain a “unitary response” that aggregates all of the transformations occurring from the nerve through to the electrode (including processing within the midbrain nuclei and any summation and filtering influencing what is recorded on the scalp). Despite the obvious simplifying assumptions of such an approach, model predictions capture many of the observed properties of ABRs and FFRs in response to simple stimuli. A slightly more elaborate model of EFRs that combines both these approaches (taking into account single-unit level phenomena such as in the model in Figure 5 as well as scalp-recording properties of the measurements as in Dau, 2003), may be considered. For instance, one recent study explored the consequences of cochlear sensitivity and selective cochlear neuropathy on the latency of simulated ABR responses (Verhulst et al., 2013). Further development, testing, and refinement will ensure that results of EFR experiments are interpreted appropriately in the context of these models. Hence, we identify this as a key area for future efforts devoted to interpreting EFR measures.

### Using envelope following responses to assess supra-threshold coding fidelity

A selective loss of lower-SR fibers would likely cause phase locking of the EFR to degrade at high sound levels, in line with the model results presented here (Figure 5B). As suggested in Figure 5, if neuropathy underlies some supra-threshold deficits, the rate of change of the EFR PLV with sound level (akin to the rate of change of ABR wave I in Furman et al., 2013) would correlate with perceptual abilities on tasks requiring analysis of the envelope of supra-threshold sounds, such as envelope ITD discrimination, spatial selective auditory attention, and related tasks. Preliminary data support this idea (Bharadwaj et al., 2013). Further experiments are needed to corroborate our hypothesis that neuropathy (especially neuropathy that preferentially affects lower-SR fibers) contributes to individual differences in the ability to analyze complex auditory scenes. The use of narrowband stimuli such as transposed tones (van de Par and Kohlrausch, 1997) with off-frequency maskers may allow for a frequency specific assessment of EFR phase locking at different CFs (i.e., at different frequency channels of the auditory pathway). If the neuropathy hypothesis proves correct, this approach may allow for a frequency-specific diagnosis of cochlear neuropathy from non-invasive physiological measures.

Despite the potential of EFRs (especially the EFR-intensity slope) for assessing cochlear neuropathy, there are some limitations. The EFR is a measure of multi-source population activity and produces scalp potentials that are different mixtures of the source activity at different scalp locations. These measures depend on the geometry of the generators, properties of the recording electrodes, the volume conductor in between, the level of unrelated electrical activity from cortex and from muscles, and other subject-specific factors (Hubbard et al., 1971; Okada et al., 1997). All of these parameters cause inter-subject variability in the absolute magnitudes of the measured EFRs. This makes interpretation of the raw EFR magnitude difficult. While phase-based metrics such as the PLV are normalized and have a straightforward interpretation (Zhu et al., 2013), their absolute strength is still influenced by the same factors. Specifically, PLV estimates are biased by the within-band SNR in the raw responses that go into the PLV computation.

This is illustrated in Figure 3B, which shows the relationship between estimated and true PLVs for simulated data (signal phase drawn from a von Mises distribution with known concentration and additive noise) as a function of SNR, under the assumptions that the noise phase in any trial is independent of the signal phase (something that can be guaranteed experimentally by jittering the stimulus presentation across trials). In Figure 3B, at sufficiently high SNRs, the estimated PLVs converge to the true PLV of the simulated signal, and are insensitive to absolute magnitudes of both signal and noise. However, at intermediate SNR values, the EFR PLV estimates are negatively biased (see Bharadwaj and Shinn-Cunningham, 2014). This has implications when trying to account for individual differences across subjects, whose raw responses may well have different SNRs. Even in within-subject comparisons, if two experimental manipulations produce responses with very different SNRs, the values of the EFR PLVs will have different biases. This is particularly important when assessing the change in PLV as a function of sound level, since high-level sounds are likely to produce stronger responses (higher SNR measurements) than low-level sounds. While an increase in response power at the stimulus modulation frequency is meaningful in itself, it is not easy to dissociate increases in PLV that result from increases in response synchrony (phase consistency) vs. from increases in response level. Minimally, using recordings in the absence of stimuli might serve to provide estimates of background noise and SNR that can then be used to extract metrics to compare fairly across subjects and conditions. How important and robust such corrections will prove depends in no small part on where on the SNR curve a particular experimental measurement falls (Figure 3B). Additional experiments are needed to characterize these effects in human listeners across different types of stimuli and experimental procedures.

Another limitation is that physiologically, the change in the basilar membrane excitation pattern with sound level also complicates the interpretation of both EFR and psychophysical results. In particular, when seeking to assess cochlear neuropathy within a specific frequency channel using PLV-level growth curves, effects of the spread of excitation are a confounding factor. Use of off-frequency maskers such as notched noise may ameliorate these effects. However, it has also been reported that at least for mid-frequency stimuli (around 1 kHz), the SSSR at the stimulus component frequency can be attenuated by noise even if the peripheral interaction between the signal and the masking noise is expected to be minimal (Gockel et al., 2012).

Alternately, EFRs can be measured in response to narrow-band stimuli with a fixed peak pressure presented at different modulation depths. For deep modulations, high-SR fibers can entrain to the modulation. At shallow modulation depths with a high sound level (carrier level), even the valleys in the signal will have sufficient energy to keep high-SR fibers saturated; thus, the strength of phase locking to shallow modulations may better reflect the contribution of lower-SR ANFs. By computing how the EFR PLV strength changes as the modulation depth is reduced, the spread-of-excitation confounds associated with manipulating the stimulus level may be avoided. Moreover, the approach of fixing the peak sound pressure and progressively decreasing the modulation depth serves to fix the point of operation on the ANF rate-level curve, so that any reduction in PLV with decreasing modulation depth can be interpreted as being related to a drop in synchrony rather than a change in average rate causing a lower SNR. The model results in Figure 5C are consistent with this notion. However, as discussed in Section Developing Quantitative Models of EFR Generators, further work is needed to relate EFR results to physiological responses of single neurons. These issues further underscore the importance of combining electrophysiological, behavioral, and modeling approaches.

## Summary and conclusions

Human listeners with normal audiometric thresholds exhibit large differences in their ability to process supra-threshold sound features. These differences can be exposed in the laboratory by challenging behavioral tasks that necessitate the use of temporal information in supra-threshold sound (e.g., segregating and selecting one auditory object out of a complex scene). While some NHT listeners seek audiological help for difficulties of this sort (a population labeled as having APD), a significant percentage of ordinary, NHT listeners recruited for psychophysical studies in the laboratory, none of whom have known hearing problems, show similar deficits under carefully designed, challenging conditions. These observations hint that perceptual problems with supra-threshold sounds are more widespread than is currently appreciated and that there may be a continuum of abilities across NHT listeners, amongst those who seek audiological help and amongst the general population.

Recent animal work shows that noise exposure and aging can result in a loss of significant proportion of ANFs without any permanent shift in detection thresholds. Moreover, this kind of neuropathy appears to preferentially affect lower-SR ANFs. Both physiological responses to AM stimuli in animals and simplistic computational model simulations suggest that lower-SR fiber loss will degrade temporal coding of sound envelopes at comfortable conversational levels, where high-SR fibers are saturated and therefore unable to entrain robustly to envelopes in input sounds.

A number of studies show that individual differences in the perception of supra-threshold sound are correlated with the strength of brainstem responses measured noninvasively on the scalp (especially SSSRs and EFRs driven by signal modulation). While the absolute strength of EFRs correlates with perceptual abilities, sensitivity of such physiological measures may be improved by using stimuli that mimic conditions akin to adverse listening conditions, such as high levels and shallow modulations. In addition, differential measures that consider how EFR phase locking changes with stimulus intensity or modulation depth may be especially sensitive when quantifying supra-threshold hearing status, helping to factor out other subject-specific differences unrelated to neuropathy. Interpretation of such measures requires assessment of cochlear function, as well as development of quantitative models of brainstem responses to establish the correspondence between population responses such as EFRs and single-unit physiology.

There are many challenges in trying to relate behavioral and EFR results to underlying physiological changes such as neuropathy, a number of which are due to gaps in current knowledge. However, converging evidence supports the hypothesis that deficits in supra-threshold coding fidelity are relatively common in the population of NHT listeners, and account for at least part of the important differences in how well these listeners can communicate in difficult everyday social settings. Here, we argue that the neuropathy seen in aging and noise-exposed animals may also be occurring in humans and that it may explain observed supra-threshold individual differences. We have also proposed some objective metrics that, based on our hypothesis, should be sensitive measures of the integrity of ANFs, allowing individual assessment of supra-threshold hearing status, and have discussed some of the limitations of the metrics. Still, there remains a large set of questions to be answered, ranging from what mechanisms cause synaptic loss that preferentially affects lower-SR fibers to what physiological or perceptual tests may be most sensitive for assessing neuropathy. We believe these questions should be addressed immediately, given the potential clinical significance of these ideas.

## Conflict of interest statement

The authors declare that the research was conducted in the absence of any commercial or financial relationships that could be construed as a potential conflict of interest.
